# FMRP: a new chapter with chromatin

**DOI:** 10.1007/s13238-014-0105-5

**Published:** 2014-10-21

**Authors:** Qingzhong He, Wei Ge

**Affiliations:** National Key Laboratory of Medical Molecular Biology & Department of Immunology, Institute of Basic Medical Sciences, Chinese Academy of Medical Sciences, School of Basic Medicine, Peking Union Medical College, Beijing, 100005 China

**Table 1:** 

EDITOR’S NOTE:	
Recently, Alpatov et al. and Zhang et al. reported the nuclear function of FMRP in replication stress-induced DNA damage response. As a well-known cytoplasmic protein functioning in pathogenesis of fragile X syndrome, FMRP’s existence and function in nucleus should be cautiously considered. Here, the authors discuss the works in an alternative perspective. They consider: 1. The subcellular fractionation strategy used to prove FMRP nuclear localization should be improved considering the nature of the protein. 2. The methyl-lysine-recognizing Agenet domains of FMRP may still needed for its neuronal function, though Alpatov et al. have elucidated that the replication stress defective FMRP mutants, which abolish its methylated histone binding function, do not alter AMPAR internalization. 3. Further investigations, such as protein interaction assessment with ATR and mapping FMRP chromatin loci with ChIP-seq, will provide deeper insight of FMRP nuclear function.	

Deficient expression of fragile X mental retardation protein (FMRP) underlies the molecular mechanism of fragile X syndrome (FXS). Traditionally, FMRP is classified as a cytoplasmic RNA-binding protein and functions as a translational repressor in the metabotropic glutamate receptor (mGluR) pathway in FXS pathogenesis. In certain contexts (Blonden et al., 2005; Feng et al., 1997; Kim et al., 2009; Van ‘t Padje et al., 2005), nuclear FMRP is also detected, yet its nuclear role remained elusive.

Recently, two groups have reported that FMRP is important in the replication stress (RS) response and may play a role in meiosis (Alpatov et al., 2014; Zhang et al., 2014).

Alpatov et al. demonstrated that in mammalian cells, H2A.X S-139 phosphorylation (γH2A.X) in response to RS, rather than double strand break (DSB), is suppressed when endogenous FMRP is down-regulated. This suppression can be rescued by exogenous expression of wild-type FMRP but not by its nucleosome-binding-deficient mutant T102A or Y103L. Similar phenomena were also observed by Zhang et al. in a *Drosophila* model, where the addition of KH domains of dFmr1 is also required for the function.

Zhang et al. found that dFmr1 increases at both the mRNA and protein levels in replication-stressed *Drosophila* S2 cells, while Alpatov et al. demonstrated that Fmrp levels in total lysate of mouse embryonic fibroblasts (MEFs) reduces slightly upon aphidicolin (APH) treatment. Using fractionation and immunofluorescence (IF) data, they both conclude that FMRP is recruited to chromatin upon RS.

Due to the nature of FMRP, such a fractionation strategy may not be suitable for researching the intracellular localization of the protein. It has been well-established that FMRP is tightly associated with the ribosome and the rough endoplasmic reticulum (RER) (Corbin et al., 1997; Feng et al., 1997; Khandjian et al., 1996), and the outer nuclear membrane (ONM) is rich in ribosome and continuous with the RER. Therefore, eliminating ONM and RER contamination in isolated chromatin fractions is a prerequisite for investigating chromatin association of FMRP; otherwise, FMRP readily appears in the nuclear fraction. In consideration of these issues, either the use of micrococcal nuclease for chromatin digestion in order to observe co-release of FMRP and nucleosomes, or immunoelectron microscopy (Feng et al., 1997) may provide a more rigorous analysis.

Both Alpatov et al. and Zhang et al. have used Leptomycin B (LPB) to facilitate IF detection of nuclear FMRP. Zhang et al. demonstrated that dFmr1 accumulate in an S2 nucleus treated with combination of hydroxyurea (HU) and LPB, but not with HU or LPB alone, and that the dFmr1 signal concentrates in the Hoechst dull staining area. In MEFs, Fmrp staining is proximal to DAPI-condensed chromocenters, reminiscent of the centromere localization of PARP-1, which has been reported to interact with FMRP (Isabelle et al., 2010).

The hypothesis that FMRP may bind histones dates back to bioinformatic analyses by Maurer-Stroh et al. (Maurer-Stroh et al., 2003). They identified that the N-terminus of FMRP contains two tandem Agenet domains of the Tudor superfamily (Maurer-Stroh et al., 2003). Subsequently, Ramos et al. prove that the Agenet domains bind methylated lysine but not arginine (Ramos et al., 2006). Destabilizing the Agenet domains does not influence the subcellular localization of FMRP cytoplasmic isoform 7, but causes its nuclear isoform 12 to lose perinucleolar localization (Ramos et al., 2006).

Alpatov et al. showed that GST-tagged FMRP Agenet domains bind native nucleosomes *in vitro*, and FMRP mutants T102A, Y103L and the disease-related mutation R138Q disrupt the interaction. The domains also enrich Kc79me2, Kc9me3, Kc27me1, Kc36me2 and Kc36me3 MLA histones significantly. Though only K79 methylation was deeply discussed by Alpatov et al., the rest may reflect other functional aspects of FMRP. K9me3 and K27me1 are well-known chromocenter markers, and may partially explain FMRP pericentromeric localization under LPB treatment (Alpatov et al., 2014); while K36 methylation serves as an active transcription marker, it may help recruit FMRP to actively transcribed genes for nascent RNA binding (Kim et al., 2009).

Zhang et al. did not consider that dFmr1 may play an essential role in H2Av phosphorylation of meiosis. However, Alpatov et al. demonstrated that Fmrp is loaded onto the pachytene chromosome in mouse spermatocyte meiosis. Further, *Fmr1* knockout mouse revealed shows reduced γH2A.X deposition at leptogene stage and excessive γH2A.X at the pachytene stage. The phenomenon is only evident in a subset of cells, which may explain that FXS patients are fertile. However the relationship between the FMRP meiotic function and FXS phenotype macroorchidism is still vague, as the phenotype is reported to be caused by aberrantly proliferated Sertoli cells (Slegtenhorst-Eegdeman et al., 1998).

Alpatov et al. report that FMRP mutants defective in the RS response do not influence the internalization of alpha-amino-3-hydroxy-5-methyl-4-isoxazole propionic acid receptor (AMPAR), indicating that FMRP chromatin function is independent of its canonical mGluR function. However, as reported by Reeve et al. (Reeve et al., 2008), dFmr1 E68K mutation (corresponding to FMRP E66K) showed a similar dorsal axonal elaborations of ventral lateral neurons to dFmr1 homozygous null mutants (Reeve et al., 2008), while by homology, FMRP E-66 is an important residue that is involved in forming salt bridge that serve to stabilize Agenet domains. This phenomenon indicates that the Agenet domains may also contribute to the FMRP neuronal function.

Using *in vitro* pull-down assays, Alpatov et al. also demonstrated that N-terminus of FMRP binds histone H3 and is dependent on lysine methylation. However, FMRP did not display a strong preference for any of the individual methylation sites. We cannot rule out the possibility that FMRP requires other intracellular protein partners, such as nucleolin, to carry out its function.

Nucleolin is a well-characterized FMRP-interacting protein. The N-terminus of FMRP interacts with recombinant nucleolin via its methylated arginine-rich region (Taha et al., 2014). Knockdown of nucleolin suppresses the elevation of γH2AX in U2OS cells upon irradiation-induced DSB damage (Kobayashi et al., 2012).

In response to RS, γH2AX formation is mediated by ATR kinase. When γH2AX formation is suppressed by FMRP down-regulation, the potential impairment of ATR kinase recruitment or activation is worth considering. The authors showed that in *FMR1* KO spermatocytes, ATR is abnormally loaded, which may partially explain the question, however further investigation is still needed.

As FMRP has been identified as chromatin-associated, chromatin immunoprecipitation followed by sequencing (ChIP-seq) is necessary to clarify its locus on chromatin (if ChIP-grade anti-FMRP is available). The data will provide deeper insight into the chromatin function of FMRP and also provide supporting information about whether or what histone modification recruits FMRP *in vivo*.

**Figure. 1:**
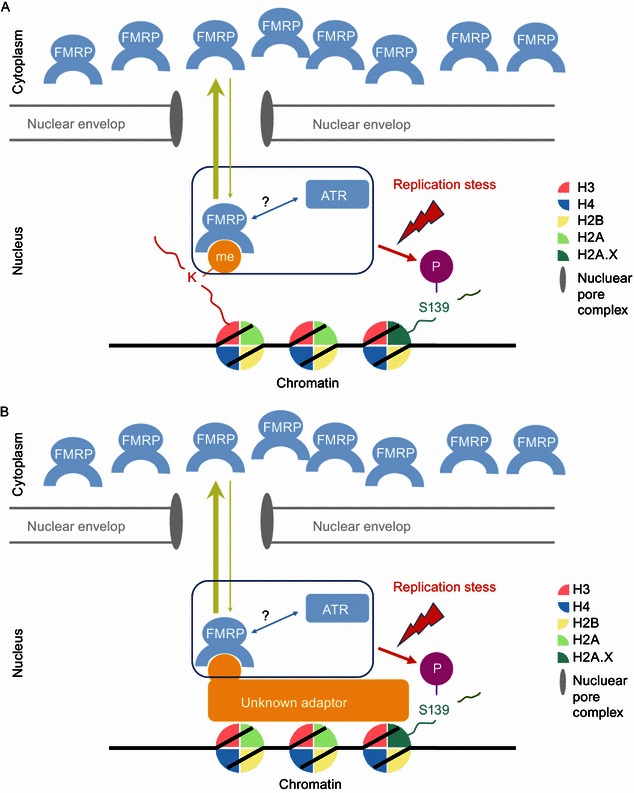
**Models of FMRP nuclear functioning**. (A) FMRP binds methylated histone lysine residues and functions in replication stress response. (B) Alternatively FMRP may cooperate with other partners via its Agenet domains to execute the same task

This year marks the 21^st^ year since the property of FMRP protein was initially characterized. As a cytoplasmic protein also functioning in chromatin, FMRP opens a new chapter of its story (Fig. 1).
